# Epigenetic-based therapies for Friedreich ataxia

**DOI:** 10.3389/fgene.2014.00165

**Published:** 2014-06-03

**Authors:** Chiranjeevi Sandi, Madhavi Sandi, Sara Anjomani Virmouni, Sahar Al-Mahdawi, Mark A. Pook

**Affiliations:** Division of Biosciences, School of Health Sciences and Social Care, Brunel University LondonUxbridge, UK

**Keywords:** Friedreich ataxia, FRDA, frataxin, FXN, GAA repeat, DNA demethylation, HDAC inhibitor, HMTase inhibitor

## Abstract

Friedreich ataxia (FRDA) is a lethal autosomal recessive neurodegenerative disorder caused primarily by a homozygous GAA repeat expansion mutation within the first intron of the *FXN* gene, leading to inhibition of *FXN* transcription and thus reduced frataxin protein expression. Recent studies have shown that epigenetic marks, comprising chemical modifications of DNA and histones, are associated with *FXN* gene silencing. Such epigenetic marks can be reversed, making them suitable targets for epigenetic-based therapy. Furthermore, since FRDA is caused by insufficient, but functional, frataxin protein, epigenetic-based transcriptional re-activation of the *FXN* gene is an attractive therapeutic option. In this review we summarize our current understanding of the epigenetic basis of *FXN* gene silencing and we discuss current epigenetic-based FRDA therapeutic strategies.

## Introduction

Friedreich ataxia (FRDA) is an autosomal recessive neurodegenerative mitochondrial disorder caused primarily by a homozygous GAA repeat expansion mutation within intron 1 of the frataxin gene (*FXN*) located on chromosome 9q21.1 (Campuzano et al., 1996). Unaffected individuals have up to 43 GAA repeats, while affected individuals have 44–1700 GAA repeats, most commonly between 600 and 900 GAA repeats (Sharma et al., 2004; Pandolfo, 2008). The effect of the GAA repeat expansion is to decrease expression of the essential and ubiquitously expressed mitochondrial protein frataxin (Campuzano et al., 1997). However, asymptomatic carriers produce about 50% frataxin levels compared to unaffected individuals (Pianese et al., 2004). Therefore, drugs that induce frataxin expression, at least to the levels of healthy carriers, would be beneficial. The *FXN* gene spans 95 kb of genomic DNA and contains seven exons, 1–5a, 5b, and 6 (Campuzano et al., 1996). The first five exons are transcribed to produce a 210 amino acid major isoform of frataxin, but rarely exon 5b can be transcribed by alternative splicing to produce a 171 amino acid protein. In addition, a recent study has identified two novel tissue specific transcript variants, encoding two isoforms of frataxin that lack the mitochondrial targeting sequence and are therefore different from the canonical transcript (Xia et al., 2012).

Reduced levels of frataxin in FRDA patients are associated with defects of iron-sulfur cluster (ISC) biosynthesis (Bradley et al., 2000), mitochondrial iron accumulation in heart and dentate nucleus (Foury and Cazzalini, 1997; Waldvogel et al., 1999; Koeppen et al., 2007), and increased susceptibility to oxidative stress (Wong et al., 1999). The main pathological effects are loss of large sensory neurons in the dorsal root ganglia (DRG) and degenerative atrophy of the posterior columns of the spinal cord, contributing to symptoms of progressive ataxia, muscle weakness, and sensory deficit. There is also pathological involvement of non-neuronal tissues, with hypertrophic cardiomyopathy a common feature, and diabetes mellitus identified in approximately 10% of FRDA patients (Schulz et al., 2009). Affected individuals generally die in early adulthood from the associated heart disease and at present there is no effective therapy for FRDA. Therefore, there is a high unmet clinical need to develop a therapy for this devastating disorder.

**Figure 1:**
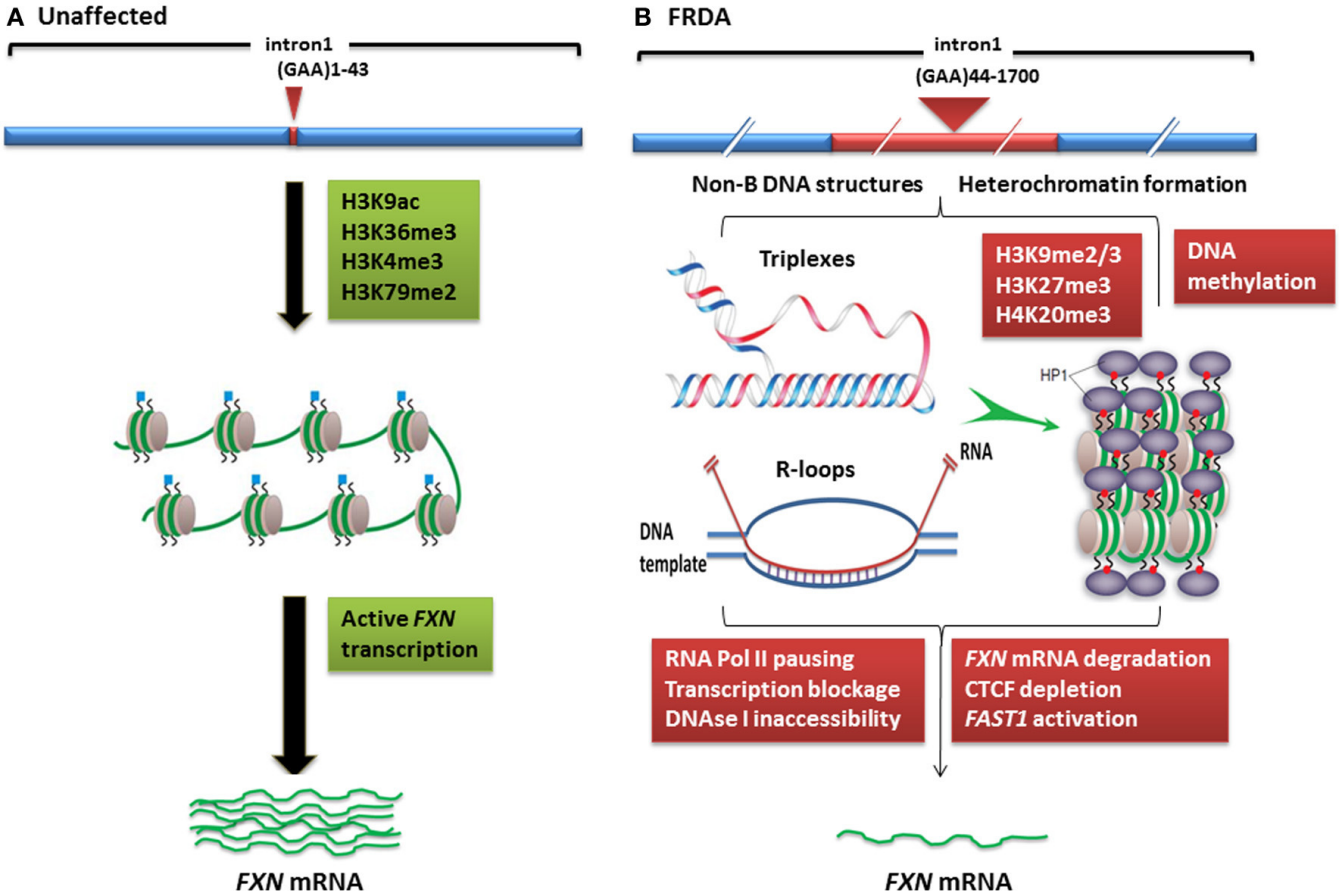
**Models of *FXN* gene silencing in FRDA. (A)** Unaffected individuals, who carry up to 43 GAA•TTC repeats, contain active histone marks of gene transcription initiation and elongation at the *FXN* promoter and intron 1 regions. **(B)** In FRDA patients, the presence of large GAA•TTC repeat expansion leads to *FXN* gene silencing by two potential mechanisms: (i) the GAA•TTC repeat may adopt abnormal non-B DNA structures (triplexes) or DNA•RNA hybrid structures (R loops), which impede the process of RNA polymerase and thus reduce *FXN* gene transcription, (ii) increased levels of DNA methylation and HP1 and significant enrichment of repressive histone marks at the *FXN* gene trigger heterochromatin formation that may lead to more pronounced *FXN* gene silencing. This image was adapted from Festenstein (2006); Wells (2008); Chan et al. (2013).

Although the exact mechanism by which the GAA repeat expansion leads to decreased frataxin expression is not known, two hypotheses have been proposed (Figure 1). Firstly, evidence from *in vitro* and cell transfection studies suggests that the GAA repeat expansion may adopt abnormal non-B DNA structures (triplexes or “sticky DNA”) or DNA•RNA hybrid structures (R-loops), which impede the process of RNA polymerase II and thus reduce *FXN* gene transcription (Grabczyk et al., 2007; Wells, 2008). Secondly, there is evidence that GAA repeat expansions can produce heterochromatin-mediated gene silencing effects (Saveliev et al., 2003). Consistent with the latter hypothesis, several FRDA disease-related epigenetic changes have been identified in the immediate vicinity of the expanded GAA repeats of the *FXN* gene and these changes will be discussed further within this review.

## Epigenetic changes associated with FRDA

Epigenetic mechanisms, including DNA methylation and hydroxymethylation, post-translational histone modifications, chromatin remodeling, and non-coding RNA effects, produce effects on gene expression without involving changes in the primary DNA sequence. The potential role of epigenetic mechanisms in FRDA disease was first suggested by the finding that long GAA repeats could suppress the expression of a nearby heterochromatin-sensitive cell surface reporter gene in transgenic mice by position effect variegation (PEV) (Saveliev et al., 2003). Further studies of FRDA cells, tissues, and mouse models have subsequently led to the identification of epigenetic changes, including DNA methylation and hydroxymethylation, histone deacetylation, and histone methylation, which may be involved in *FXN* gene silencing in FRDA (Table 1).

**Table 1:** **Epigenetic changes in tissues, cells, and animal models of FRDA**.

**Chromatin change^a^**	**Location**	**FRDA tissue, cell, or animal model**	**References**
5mC ↑	GAA upstream	FRDA patient tissues, primary lymphocytes, lymphoblasts, FXN YAC transgenic mice	Greene et al., 2007; Al-Mahdawi et al., 2008, 2013; Evans-Galea et al., 2012
5hmC ↑	GAA upstream	FRDA patient tissues	Al-Mahdawi et al., 2013
H3K4me2/3 ↓	*FXN* promoter and exon 1	Lymphoblasts	Punga and Buhler, 2010; Kim et al., 2011; Kumari et al., 2011
	GAA upstream	Lymphoblasts	Kim et al., 2011; Kumari et al., 2011
	GAA downstream	Lymphoblasts	Kim et al., 2011; Kumari et al., 2011
H3K9me2/3 ↑	*FXN* 5'UTR/promoter	Primary fibroblasts, lymphoblasts	De Biase et al., 2009; Kim et al., 2011
	GAA upstream	Lymphoblasts, *FXN* YAC transgenic mice, KIKI mice	Herman et al., 2006; Al-Mahdawi et al., 2008; Rai et al., 2008; Punga and Buhler, 2010; Kim et al., 2011; Kumari et al., 2011; Chan et al., 2013
	GAA downstream	FRDA patient tissues, *FXN* YAC transgenic mice, lymphoblasts	Herman et al., 2006; Al-Mahdawi et al., 2008; Punga and Buhler, 2010; Kim et al., 2011; Kumari et al., 2011; Chan et al., 2013
H3K27me3 ↑	*FXN* 5'UTR/promoter	Primary fibroblasts, lymphoblasts	De Biase et al., 2009; Kim et al., 2011
	GAA upstream	Lymphoblasts	Kim et al., 2011; Chan et al., 2013
	GAA downstream	Lymphoblasts	Kim et al., 2011; Chan et al., 2013
H3K36me3 ↓	GAA upstream	Lymphoblasts	Punga and Buhler, 2010; Kim et al., 2011; Kumari et al., 2011
	GAA downstream	Lymphoblasts	Punga and Buhler, 2010; Kumari et al., 2011; Kim et al., 2011
H3K79me2 ↓	GAA upstream	Lymphoblasts	Kim et al., 2011
	GAA downstream	Lymphoblasts	Kim et al., 2011
H4K20me3 ↑	GAA upstream	Lymphoblasts	Kim et al., 2011
	GAA downstream	Lymphoblasts	Kim et al., 2011
H3K9ac ↓	*FXN* promoter	FRDA patient brain tissue, lymphoblasts	Al-Mahdawi et al., 2008; Kumari et al., 2011
	GAA upstream	FRDA patient tissues, lymphoblasts, *FXN* YAC transgenic mice, KIKI mice	Herman et al., 2006; Al-Mahdawi et al., 2008; Kumari et al., 2011
	GAA downstream	FRDA patient tissues, lymphoblasts, *FXN* YAC transgenic mice	Herman et al., 2006; Al-Mahdawi et al., 2008; Kumari et al., 2011
H3K14ac ↓	*FXN* promoter	FRDA patient tissues	Al-Mahdawi et al., 2008
	GAA upstream	Lymphoblasts, KIKI mice	Rai et al., 2008; Kumari et al., 2011
	GAA downstream	Lymphoblasts, *FXN* YAC transgenic mice	Kumari et al., 2011
H4K5ac ↓	*FXN* promoter	Lymphoblasts	Kumari et al., 2011
	GAA upstream	Lymphoblasts, KIKI mice	Herman et al., 2006; Rai et al., 2008; Kumari et al., 2011
	GAA downstream	FRDA patient tissues, *FXN* YAC transgenic mice, lymphocytes	Al-Mahdawi et al., 2008; Rai et al., 2008; Kumari et al., 2011
H4K8ac ↓	GAA upstream	Lymphoblasts, FRDA patient tissues, KIKI mice	Herman et al., 2006; Al-Mahdawi et al., 2008; Rai et al., 2008; Kumari et al., 2011
	GAA downstream	Lymphoblasts, FRDA patient tissues	Herman et al., 2006; Kumari et al., 2011
H4K12ac ↓	*FXN* promoter	Lymphoblasts	Herman et al., 2006
	GAA upstream	Lymphoblasts, FRDA patient tissues, *FXN* YAC transgenic mice	Herman et al., 2006; Al-Mahdawi et al., 2008
	GAA downstream	Lymphoblasts, FRDA patient tissues, *FXN* YAC transgenic mice	Herman et al., 2006; Al-Mahdawi et al., 2008
H4K16ac ↓	*FXN* promoter	Lymphoblasts	Rai et al., 2008; Kumari et al., 2011
	GAA upstream	Lymphoblasts, FRDA patient tissues, *FXN* YAC transgenic mice, KIKI mice	Herman et al., 2006; Al-Mahdawi et al., 2008; Rai et al., 2008; Kumari et al., 2011
	GAA downstream	Lymphoblasts, FRDA patient tissues, *FXN* YAC transgenic mice, KIKI mice	Herman et al., 2006; Al-Mahdawi et al., 2008; Rai et al., 2008; Kumari et al., 2011

a ↓, reduced; ↑, increased; H, histone; K, lysine; me2, dimethylation; me3, trimethylation; ac, acetylation.

### DNA methylation and hydroxymethylation

DNA methylation is carried out by DNA methyltransferase (DNMT) enzymes, which catalyze the conversion of cytosine to 5'-methylcytosine (5mC), predominantly within CpG dinucleotides (Robertson, 2001). In mammals, the DNMT family includes three functional proteins: DNMT1 preferentially methylates hemi-methylated DNA and is thus responsible for methylation during DNA replication (Pradhan et al., 1999), while DNMT3a and DNMT3b have an equal preference for hemi-methylated and non-methylated DNA and so have been classified as *de novo* methyltransferases (Okano et al., 1999). Studies investigating the DNA methylation profiles of transcriptionally silenced genes have revealed a strong correlation between promoter DNA methylation and transcriptional silencing. However, it has also been reported that intragenic DNA methylation can contribute to transcriptional gene silencing (Lorincz et al., 2004). More recent studies have revealed the existence of an alternative modification, 5'-hydroxymethylcytosine (5hmC), which is formed by oxidation of 5mC by ten-eleven translocation (TET) enzymes (Kriaucionis and Heintz, 2009; Tahiliani et al., 2009). 5hmC may either be an intermediate in the removal of 5mC by an active demethylation process (Guo et al., 2011) or it may be an epigenetic modification in its own right, regulating chromatin or transcriptional factors (Szulwach et al., 2011). Therefore, it is possible that 5hmC may prove to be involved in epigenetic-based disease mechanisms, such as those proposed for FRDA.

**Figure 2:**
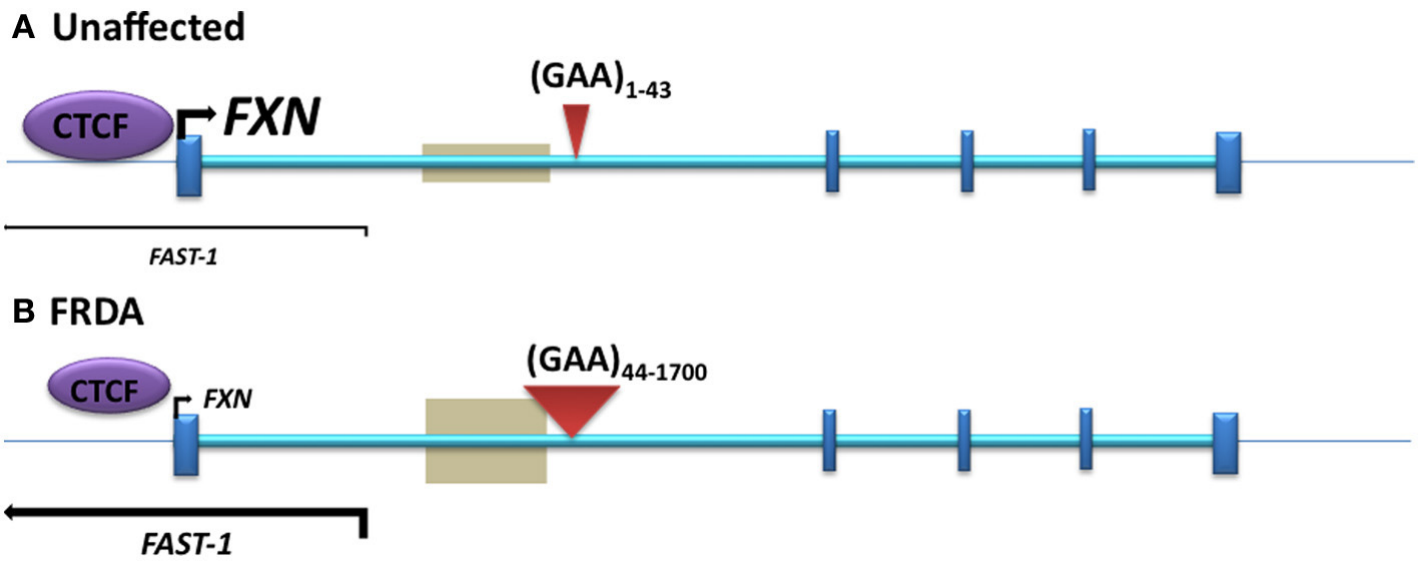
**The position of DNA methylation, hydroxymethylation, and CTCF binding sites within the *FXN* gene. (A)** Unaffected: normal-sized GAA repeat **(B)** FRDA: GAA repeat expansion. Gray boxes represent regions of disease-associated DNA methylation and hydroxymethylation. Arrow marks represent the directions and levels of transcription for *FXN* and *FAST-1*. Blue bars represent exons of the *FXN* gene. Red triangles indicate GAA repeats within intron 1 of the *FXN* gene.

Initial investigations of DNA methylation within the *FXN* gene by Usdin and colleagues revealed hypermethylation of specific CpG sites upstream of the GAA repeat sequence in FRDA patient-derived lymphoblastoid cells compared to cells derived from unaffected individuals (Greene et al., 2007). In particular, three out of 15 CpG residues that span a 700 bp region upstream of the GAA repeat were found to be unmethylated, or sparsely methylated, in unaffected cells, but highly methylated in FRDA cells. Studies by our laboratory also revealed significantly increased DNA methylation at the same upstream GAA repeat region in FRDA patient autopsy brain, heart, and cerebellum tissues, with similar findings in tissues of FRDA YAC transgenic mice (Al-Mahdawi et al., 2008). Subsequently, a study of FRDA patient blood samples showed that the degree of DNA methylation at the *FXN* upstream GAA repeat region correlates with the length of the GAA repeats and inversely correlates with the age of disease onset (Castaldo et al., 2008). Furthermore, analysis of blood and buccal cells from a large cohort of FRDA patients showed that the level of DNA methylation in FRDA patients is significantly elevated in the same upstream GAA repeat region and inversely correlated with *FXN* expression levels (Evans-Galea et al., 2012). More recently, our laboratory has shown that, for at least one CpG site within the upstream GAA repeat region, the increased level of DNA methylation predominantly comprises 5hmC rather than 5mC (Al-Mahdawi et al., 2013). Thus, FRDA can now be grouped together with other TNR expansion diseases in which an association with DNA methylation and hydroxymethylation has been observed (Table 1, Figure 2).

### Post-translational histone modifications

The nucleosome consists of an octamer of four pairs of histone proteins, H2A, H2B, H3, and H4, surrounded by 147 bp of DNA (Kouzarides, 2007). Histone proteins contain a globular C-terminal domain and an unstructured N-terminal tail, which can contain many different modified residues. Histone acetylation at lysine residues is regulated by two distinct families of enzymes with opposing actions, histone acetyltransferases (HATs) and histone deacetylases (HDACs). Similarly, histone lysine methylation is controlled by histone methyltransferases (HMTases) and histone demethylases (HDMs), which have been linked to a number of cellular processes including transcriptional activation and repression (Kouzarides, 2007). HATs are diverse set of enzymes that are grouped into two different families based on their catalytic domains: Gcn5 N-acetyltransferases (GNATs, e.g., Gcn5 and PCAF) and MYST HATs (e.g., Morf and Ybf2) (Kimura et al., 2005). Each HAT is usually capable of modifying several different histone residues. Eighteen different HDACs have been identified in mammals and these have been divided into four classes. Class I consists of HDACs 1, 2, 3, and 8, which are similar to yeast RPD3 deacetylase. Class II consists of HDACs 4, 5, 7, and 9, which have homology to the yeast HDAC *Had-1* gene. Class III HDACs, also known as sirtuins, which have homology to the Yeast *Sir2* gene, include sirtuin 1–7. Lastly, class IV HDACs consists of only HDAC 11. HMTases are divided into lysine-specific and arginine-specific groups. They have great specificity and usually modify only one particular histone residue (Kouzarides, 2007). The HDMs are classified into two distinct enzyme families: the nuclear amine oxidase homologs (e.g., LSD1) and the JmjC-domain proteins (e.g., JHDM1) (Kouzarides, 2007).

In mammals, heterochromatin formation and gene silencing is associated with hypoacetylation of certain histone residues, particularly H3K9, together with increased methylation of histone residues, including H3K9me2, H3K9me3, H3K27me3, and H4K20me3 (Kourmouli et al., 2004; Martin and Zhang, 2005; Jenuwein, 2006). Such histone modifications have now been identified within the *FXN* gene in FRDA cells, tissues, and mouse models, predominantly at the region immediately upstream of the expanded GAA repeats, indicating that the *FXN* gene is subject to heterochromatin silencing (Sandi et al., 2013) (Table 1, Figure 1). Histone modifications at the *FXN* locus were first identified by Gottesfeld and colleagues, who reported lower levels of several acetylated H3 and H4 lysine residues, together with increased H3K9me2 and H3K9me3 in the upstream GAA regions of FRDA lymphoblastoid cells (Herman et al., 2006). Usdin and colleagues then reported increased H3K9me2 levels within *FXN* intron 1 in FRDA lymphoblastoid cells (Greene et al., 2007) and our laboratory reported changes of histone modifications at the *FXN* promoter, upstream, and downstream GAA repeat regions in FRDA patient and YAC transgenic mouse brain tissues (Al-Mahdawi et al., 2008). Subsequently, Bidichandani and colleagues reported that FRDA patient fibroblasts have significantly higher levels of H3K9me3 and H3K27me3 at the *FXN* 5' UTR region, coupled with elevated levels of heterochromatin protein 1 (HP1), compared to normal fibroblasts (De Biase et al., 2009). More recently, Chan and colleagues have identified increased H3K9me3 and H3K27me3 at the upstream and downstream GAA repeat regions in FRDA lymphoblasts (Chan et al., 2013).

In contrast, histone modifications such as H3K4me3, H3K36me3, and H3K79me3 are associated with a more open chromatin state and active gene expression. H3K4me3 is particularly associated with initiation of gene transcription, while H3K36me3 and H3K79me3 are associated with elongation of gene transcription. Recent studies have shown decreased levels of H3K36me3 and H3K79me3 at the upstream and downstream GAA repeat regions of the *FXN* gene in FRDA cells, indicating that there is a defect in transcription elongation (Punga and Buhler, 2010; Kim et al., 2011; Kumari et al., 2011). Decreased levels of H3K4me3 have also been identified at the upstream GAA repeat region, but not at the promoter region, which suggests a more pronounced defect of the post-initiation and elongation stages of *FXN* gene expression rather than an early transcription initiation defect (Punga and Buhler, 2010; Kim et al., 2011; Kumari et al., 2011) (Figure 1). In summary, there is good evidence that reduction of frataxin protein expression in FRDA is primarily caused by GAA repeat expansion-induced transcriptional silencing, which is associated with specific post-translational histone modifications.

### Antisense transcription and CTCF binding

Antisense RNA transcripts, which play a role in regulation of gene expression, have previously been associated with TNR expansion diseases such as Huntington disease (HD) (Chung et al., 2011), FRAXA (Ladd et al., 2007; Khalil et al., 2008), DM1 (Cho et al., 2005), SCA7 (Sopher et al., 2011), and SCA8 (Moseley et al., 2006). Furthermore, the CCCTC-binding factor (CTCF) protein, which can prevent the spreading of DNA methylation (Filippova et al., 2005; Engel et al., 2006), has binding sites in the repeat expansion flanking regions of several TNR disorders, such as FRAXA (Ladd et al., 2007), DM1 (Filippova et al., 2001), and SCA7 (Libby et al., 2008), and loss of CTCF binding at the DM1 CTG expansion is associated with the spread of heterochromatin and DNA methylation (Cho et al., 2005). Bidichandani and colleagues have reported significantly increased levels of a frataxin antisense transcript 1 (*FAST1*) in FRDA fibroblast cells, associated with depletion of CTCF binding at the 5' UTR region of the *FXN* gene (Figure 2), suggesting involvement of these factors in the heterochromatin and *FXN* gene silencing processes of FRDA disease (De Biase et al., 2009). More recently, our laboratory has also reported increased expression of *FAST1* in FRDA mouse fibroblasts (Sandi et al., 2014) and reduced CTCF binding at the 5' UTR region of the *FXN* gene in FRDA cerebellum tissue (Al-Mahdawi et al., 2013). Systematic genome-wide mapping by ChIP analysis has shown that CTCF binds to tens of thousands of genomic sites, often distal to transcription start sites (TSS) of genes, but also at promoter and enhancer sequences (Barski et al., 2007; Holwerda and De Laat, 2013). Thus, CTCF can direct the interaction of tissue-specific enhancers with different neighboring promoters resulting in the regulation of gene expression at a particular locus (Shen et al., 2012). The association of CTCF binding at the 5' UTR region of the *FXN* gene with high levels of *FXN* expression and low levels of *FAST1* expression indicates that such CTCF control mechanisms might be in operation at the *FXN* locus. In a large proportion of cases, control of CTCF binding has been shown to be sensitive to DNA methylation (Wang et al., 2012). However, this is not likely to be the case for FRDA, because FRDA-specific changes in DNA methylation have not been detected at the CTCF-binding site within the 5' UTR region of the *FXN* gene (Al-Mahdawi et al., 2008; De Biase et al., 2009). Therefore, further consideration of antisense transcription, CTCF binding, and other associated factors are needed, since they are likely to be highly relevant to the development of an epigenetic-based therapy for FRDA.

## Epigenetic-based therapies for FRDA

### DNA demethylation therapies

Since FRDA is associated with increased levels of DNA methylation, one can propose the use of DNA demethylating agents to potentially activate a silenced *FXN* gene. DNA demethylating agents are generally divided into nucleoside analog DNMT inhibitors and non-nucleoside analog DNMT inhibitors. Nucleoside analog DNMT inhibitors, including 5-azacytidine (5-aza-CR or Vidaza), 5-aza-2'-deoxycytidine (5-aza-CdR or Decitabine), and Zebularine, are analogs of cytosine. 5-aza-CdR is an FDA-approved drug that is used to treat leukemia patients (Jain et al., 2009). Furthermore, treatment of lymphoblastoid cells from FRAXA patients with 5-aza-CdR, either alone (Chiurazzi et al., 1998) or in combination with HDAC inhibitors (Chiurazzi et al., 1999), efficiently reverses the FMR1 promoter hypermethylation and restores mRNA and protein levels to normal. Such studies have led to the consideration of DNA demethylating agents as a potential therapy for neurodegenerative disorders. Thus, far, there have been no reports of describing the use of DNA demethylating agents as a therapeutic approach for FRDA, mainly due to the lack of evidence for a causal link between DNA methylation and *FXN* gene silencing. However, DNA demethylating agents, such as 5-aza-CdR, Zebularine, or the oligonucleotide antisense inhibitor of DNMT1, MG98 (Plummer et al., 2009), may be considered as FRDA therapeutic options in the future.

### HDAC inhibitors

**Figure 3:**
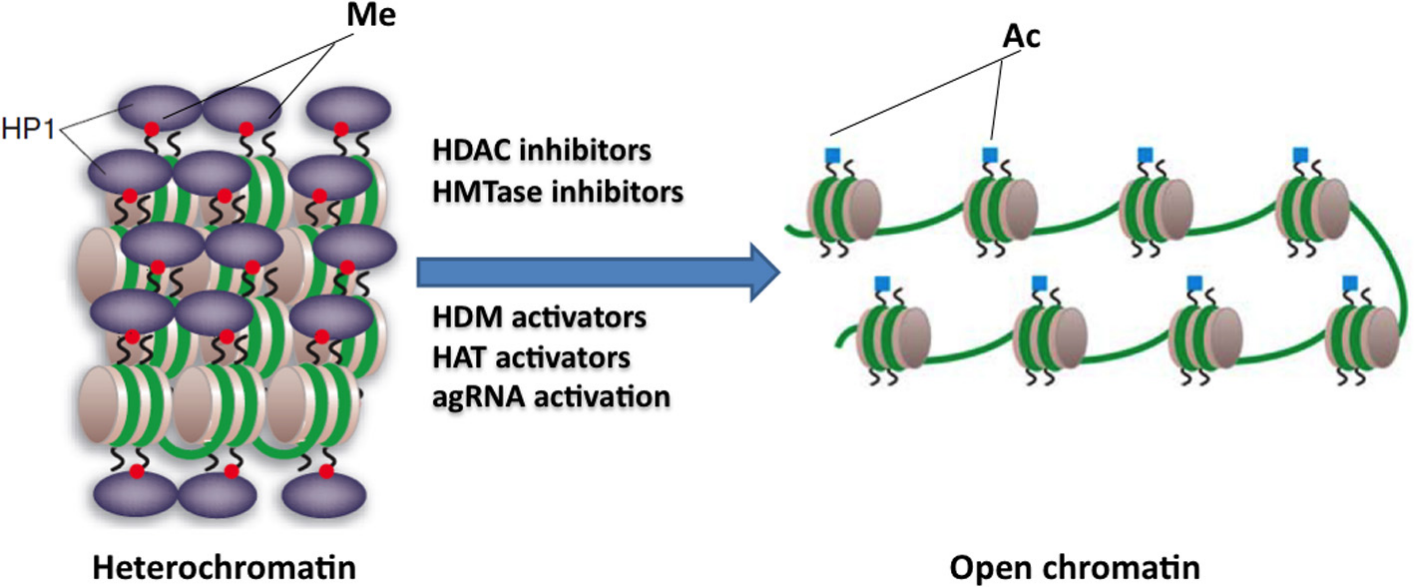
**Potential epigenetic-based therapies for FRDA**. Large GAA•TTC repeats in FRDA patients are associated with heterochromatin mediated *FXN* gene silencing. The use of specific HDAC inhibitors, HDM activators, HAT activators, HMTase inhibitors, or agRNA activation may reverse the heterochromatin to a more open chromatin structure, and may thus lead to active *FXN* gene transcription. This image was adapted from Festenstein (2006); Chan et al. (2013).

HDAC inhibitors can affect transcription by increasing acetylation of histones, transcription factors, and other proteins that regulate transcription (Butler and Bates, 2006). In view of the recent identification of histone acetylation changes at the *FXN* gene in FRDA, it has been proposed that the reversal of such histone modifications could represent a useful therapeutic approach for FRDA (Figure 3) (Festenstein, 2006; Herman et al., 2006). An initial study to screen for frataxin-increasing compounds by Sarsero and colleagues first demonstrated a small effect of the general HDAC inhibitor sodium butyrate on *FXN* gene activity using an EGFP reporter cell line (Sarsero et al., 2003). Subsequently, Gottesfeld and colleagues treated FRDA lymphoblastoid cells using a selection of commercially available HDAC inhibitors. They revealed that only the benzamide compound BML-210 produced a significant increase of *FXN* mRNA expression (Herman et al., 2006), although other HDAC inhibitors showed a more pronounced increase of histone acetylation without any increase in *FXN* expression, indicating a degree of compound specificity for *FXN* gene silencing (Table 2). An analog of BML-210, designated **4b**, was then synthesized and was shown to directly modulate the histones associated with the *FXN* gene to increase *FXN* mRNA expression in FRDA primary lymphocytes (Herman et al., 2006). Compound **4b** has subsequently been used to demonstrate amelioration of the HD phenotype in R6/2 mice without any discernible toxicity (Thomas et al., 2008). Further development of HDAC inhibitors by the Gottesfeld lab and the Repligen Corporation (Waltham, Massachusetts) identified three 2-aminobenzamide compounds, designated **106, 136**, and **109**, which can each significantly increase *FXN* mRNA and frataxin protein levels in FRDA cells with only a small effect on unaffected control cells (reviewed in Soragni et al., 2012). The exact mechanism of action of the 2-aminobenzamide HDAC inhibitors in FRDA is not known. However, compound **106** has been shown to act as selective slow-on, slow-off, tight-binding inhibitor of class I HDACs, with a preference for inhibition of HDAC3 (Chou et al., 2008; Xu et al., 2009). Compounds **106, 136**, and **109** have undergone investigations to determine safety, efficacy and pharmacokinetic profile in short-term treatments of FRDA patient derived cells and KIKI knock-in mice (Chou et al., 2008; Rai et al., 2008, 2010; Soragni et al., 2008; Xu et al., 2009), and a long-term treatment in YG8R YAC transgenic mice (Sandi et al., 2011) (Table 2). Compound **109**, which emerged as the most promising compound for FRDA treatment, has now been assessed in a phase I clinical trial as RG2833 (Soragni et al., 2012) and further related compounds are now under development.

**Table 2:** **HDAC inhibitor preclinical studies of FRDA cells and mice**.

**HDAC inhibitor**	**FRDA test system**	***FXN* mRNA change**	**Frataxin protein change**	**Change in *FXN* histone marks**	**References**
Sodium butyrate	*FXN*-EGFP reporter cell line		1.16 fold ↑ (*FXN*-EGFP)		Sarsero et al., 2003
Trichostatin A	Lymphoblasts	No change, 1.2 fold ↑			Herman et al., 2006
SAHA	Lymphoblasts	No change, 1.6 fold ↑			Herman et al., 2006
Oxamflatin	Lymphoblasts	1.5 fold ↑			Herman et al., 2006
BML-210	Lymphoblasts	2.2 fold ↑			Herman et al., 2006
4b	Primary lymphocytes	2.3 fold ↑	3–3.5 fold ↑	3 fold ↑ (H3K14ac, H4K5ac, and H4K12ac)	Herman et al., 2006
	Lymphoblasts	2.5 fold ↑			
106	Primary lymphocytes	1.7–3.6 fold ↑			Rai et al., 2008, 2010; Soragni et al., 2008; Sandi et al., 2011
	Lymphoblasts	2.6 fold ↑			
	KIKI mice	1.2–1.6 fold ↑	1.4 fold ↑	1.2–1.5 fold ↑ (H4K5ac, H4K8ac, H4K16ac, and H3K14ac)	
	*FXN* transgenic mice	No change	1.6 fold ↑	1.8–2 fold ↑ (H4K5ac)	
136	Primary lymphocytes	1.2–2 fold ↑			Rai et al., 2010; Sandi et al., 2011
	KIKI mice	1.2 fold ↑		1.2–1.5 fold ↑ (H3K14ac and H4K5ac)	
	*FXN* transgenic mice	No change	2 fold ↑	No change	
109	Primary lymphocytes	2–8 fold ↑	2–3.6 fold ↑		Rai et al., 2010; Sandi et al., 2011
	KIKI mice	1.2–1.3 ↑		1.2–1.5 fold ↑ (H3K14ac and H4K5ac)	
	*FXN* transgenic mice	No change	2.6 fold ↑	1.2–8 fold ↑ (H3K9ac, H4K5, and H4K12ac)	
Nicotinamide	Lymphoblasts	1.06–1.8 fold ↑		2 fold ↑ H3ac 1.8 fold ↑ H4ac	Chan et al., 2013
	Primary lymphocytes	4.5 fold ↑		2.9 fold ↓ H3K9me3 and 1.56 fold ↓ H3K27me3	
	*FXN* YAC transgenic mice	1.3–1.8 fold ↑		2.1 fold ↓ H3K9me3 and 1.6 fold ↓ H3K27me3	
C5	Lymphoblasts			2–3 fold ↑ (H3K9ac and H4K8ac)	Lufino et al., 2013
	Primary lymphocytes	1.5–2 fold ↑			Lufino et al., 2013

Recently, the class III HDAC inhibitor nicotinamide (Ghosh and Feany, 2004) has been shown to increase frataxin expression and decrease H3K9me3 and H3K27me3 at the *FXN* gene in FRDA cells and mouse models and this compound is now in early stage clinical trials (Chan et al., 2013) (Table 2). Furthermore, other HDAC inhibitors such as sirtinol (Ota et al., 2006), splitomicin (Biacsi et al., 2008), LBH589 (Garbes et al., 2009), and oxamflatin (Kim et al., 1999) have shown positive effects in other diseases including cancer and/or neurodegenerative disorders and these compounds may also be considered for future FRDA therapy. A number of research groups are also currently screening for novel frataxin-increasing compounds and one group has identified a novel HDAC inhibitor, designated **C5**, which upregulates *FXN* expression in FRDA patient primary lymphocytes and increases H3K9ac and H4K8ac in FRDA lymphoblasts (Lufino et al., 2013) (Table 2). Interestingly, it has also recently been reported that resveratrol, a sirtuin activator, can produce a marked increase in frataxin expression in a *FXN*-EGFP reporter cell line and in a FRDA mouse model (Li et al., 2013). Therefore, further studies are required to dissect the interplay of HDACs and compounds that modulate HDAC activity in the direct or indirect control of *FXN* gene expression. Epigenetic-based therapies for FRDA are also likely to have other off-target effects, which do not directly affect *FXN* gene regulation, but which may nevertheless have other positive or negative effects on FRDA disease. For example, recent studies have demonstrated that frataxin deficiency in a conditional knockout mouse model triggers SIRT3 inhibition, resulting in marked hyperacetylation of numerous cardiac mitochondrial proteins (Wagner et al., 2012) that may lead to an increased sensitivity to oxidative stress. This finding suggests that the compounds that specifically activate SIRT3 could be a beneficial FRDA therapy.

### HMTase inhibitors

HMTase inhibitors, which may induce a more open chromatin structure at the *FXN* gene, are now also being considered for FRDA therapy (Figure 3). For example GSK126 has been identified as a potent and highly specific inhibitor of EZH2, a major component of the Polycomb repressor complex 2 (PRC2) (McCabe et al., 2012). EZH2 trimethylates lysine 27 on the tail of histone H3 (H3K27) and has been linked to the repression of several genes, thus, inhibition of this repressive histone mark at active genes could have a beneficial therapeutic outcome. High throughput biochemical screening has revealed genome-wide loss of H3K27 methylation and activation of previously silenced genes with minimal off-target effects following treatment with GSK126 (McCabe et al., 2012). In addition, small cell lung cancer (SCLC) cells treated with GSK126 showed substantial inhibition of cellular growth and this inhibition was concomitant with the reduced levels of H3K27me3 (Sato et al., 2013). These findings have highlighted the fact that inhibition of EZH2 activity by small molecule inhibitors, like GSK126, has a therapeutic benefit. In FRDA, increased levels of H3K27me3 have been reported in lymphoblastoid cells and fibroblast cells (De Biase et al., 2009; Kim et al., 2011; Chan et al., 2013) (Table 1). Therefore, this compound, which can inhibit such repressive marks, could have a beneficial FRDA therapeutic effect by reactivating *FXN* gene transcription.

Other studies have shown that H3K9me2 can be erased from the promoters of reactivated tumor suppressor genes following treatment with DNMTs and/or HDAC inhibitors (McGarvey et al., 2006). Therefore, compounds which can reduce H3K9me2 levels are an attractive option where gene silencing involves increased H3K9me2 levels, as seen with FRDA (Sandi et al., 2013) (Table 1). This has led to the development of various compounds that can inhibit the G9a activity directly or indirectly. The initial screening of 125,000 compounds from a chemical library has identified BIX-01294 as a potent and specific inhibitor of G9a (Kubicek et al., 2007). BIX-01294 binds to the SET domain of GLP in the same groove at which the target lysine (H3K9) binds. This prevents the binding of the peptide substrate and, consequently, the deposition of methylation marks at H3K9 (Chang et al., 2009). Recent studies in FRDA lymphoblastoid cells using the HMTase inhibitor, BIX-01294, identified a significant reduction in H3K9me2/me3, but without increasing the *FXN* mRNA levels, suggesting a possible redundant role for H3K9me2/me3 in *FXN* gene silencing (Punga and Buhler, 2010). In contrast, Chan and colleagues have recently identified nicotinamide-induced reactivation of the *FXN* gene in association with decreased H3K9me3 and H3K27me3 (Chan et al., 2013). Recently, a new inhibitor for G9a and GLP, UNC0638, has been identified (Vedadi et al., 2011). Treatment of a variety of cell lines with UNC0638 resulted in lower global H3K9me2 levels and activated several previously silenced genes *in vitro* (Vedadi et al., 2011). In addition, treatment of U2OS and HeLa cells with UNC0638 has shown that it specifically inhibits H3K9me2, with minimal effect on H3K27me3 and H4Kme3 levels (Machleidt et al., 2011). ERG-associated protein with SET domain (ESET), a novel histone H3K9 methyltransferase, has been shown to mediate histone methylation (Rea et al., 2000). It is also proposed that ESET may have a role in epigenetic silencing of neuronal genes through its HMTase activity (Li et al., 2006). Importantly, the ESET levels were significantly elevated in HD patients and in R6/2 transgenic mice (Ryu et al., 2006). Subsequent treatment with mithramycin (or in combination with cystamine), resulted in substantial reduction of H3K9me3 and significantly improved the behavioral and neuropathological phenotype in R6/2 and 82Q mice of HD (Ryu et al., 2006; Stack et al., 2007). Mithramycin is a clinically approved guanosine-cytosine rich DNA antitumor antibiotic that interferes with the DNA binding of the Sp family transcription factors, but its effects in FRDA have not yet been studied. Ongoing preclinical studies of cell and mouse models using such HMTase inhibitors will determine if any of these compounds can be considered suitable to progress to FRDA clinical studies, as is the case now for HDAC inhibitors.

### Antigene RNA-based therapies

Antigene RNAs (agRNAs) are small duplex RNAs of 19 bp in length that target gene promoters. Depending on the target sequence and cell type, agRNAs can either silence (Janowski et al., 2006) or activate the gene transcription (Janowski et al., 2007). Since agRNAs target in a sequence specific manner, it may be possible to modulate agRNAs to activate gene expression in disease-associated genes where gene activation is essential, as with FRDA. Importantly, agRNAs can target both sense or antisense strands, and coding or non-coding RNA transcripts (Watts et al., 2010). Therefore, the use of agRNA to activate the *FXN* gene by targeting the *FXN* promoter or the *FAST1* transcript may prove useful, since both mechanisms may be involved in reversing *FXN* gene silencing and thus ameliorating FRDA disease. However, as with any macromolecule-based therapy, the major problem of how to achieve effective delivery to cells will first have to be overcome.

## Potential risks of epigenetic therapies

Epigenetic therapies to date have primarily focused on the use of DNA hypomethylating agents and HDAC inhibitors. The major risk associated with any epigenetic therapy is the potential lack of specificity. To the best of our knowledge it is not yet possible to silence or reactivate specific genes through use of DNMT inhibitors or HDAC inhibitors. Due to the general effects of these compounds, they tend to disrupt global DNA methylation and histone acetylation status. Genome-wide loss of DNA methylation has been shown to produce a high incidence of lymphomas in mouse models (Gaudet et al., 2003), which raises the concern of potential tumor formation in patients given DNA hypomethylating treatments. HDAC inhibitors also have off-target effects that may be difficult to detect, especially if they appear years after the initial therapy. This is important, because most FRDA patients are diagnosed before adulthood. Therefore, prolonged HDAC inhibitor treatment in childhood may induce tumor formation, for example, that is only apparent later in adulthood. However, studies to date show that HDAC inhibitors are actually reasonably well tolerated in the short term. They appear to have less toxicity when used for cancer treatment than classical cytotoxic chemotherapeutic agents, but they are still prone to induce primary clinical toxicities such as cardiac side effects, nausea, vomiting, fatigue, and reduced blood cell counts (Subramanian et al., 2010). The clinical use of HMTase inhibitors has not advanced as far as the use of HDAC inhibitors, but HMTase inhibitors are also expected to induce extensive gene expression changes, and hence potential off-target effects. Having said this, use of the HMTase inhibitor GSK126 for the treatment of lymphoma has been shown to exert minimal off-target effects despite genome-wide changes in H3K27 methylation, which is somewhat encouraging (McCabe et al., 2012).

## Conclusions

Due to the identification of several associated epigenetic marks, FRDA can now be considered as an epigenetic disease and drug treatments are being developed to target these epigenetic changes in attempts to ameliorate the disease phenotype. However, epigenetic-based therapies are generally non-specific, with off-target effects, and the development of drugs to more specifically target the *FXN* locus may require further consideration. Epigenetic-based therapies for FRDA will also benefit from further understanding of how *FXN* gene expression is controlled. For example, it has been recently reported that two transcription factors, SRF and TFAP2, directly bind to the promoter region of the human *FXN* gene and alter frataxin mRNA and protein levels (Li et al., 2010). Small non-coding RNAs, such as microRNAs, have also been implicated in several neurodegenerative disorders including AD (Cogswell et al., 2008), HD (Lee et al., 2011), and FRDA (Mahishi et al., 2012). An elevated level of miR-886-3p in FRDA is associated with the downregulation of the *FXN* gene and the use of anti-miR-886-3p or the HDAC inhibitor **4b** alone has been shown to partially reverse *FXN* gene repression by reducing miR-886-3p levels (Mahishi et al., 2012; Bandiera et al., 2013). Therefore, it would be interesting to investigate the combined effect of anti-microRNA and HDAC inhibitor compounds in the activation of *FXN* gene transcription. In general, it may also be useful to simultaneously administer two or more epigenetic-based drugs to examine synergistic treatment effects. In addition, to identify novel epigenetic-based FRDA therapeutic compounds, various drug-screening systems, cell and animal models have been developed and are currently being utilized by several labs (see review Martelli et al., 2012). Overall, considering the rapid progress that has been made to take HDAC inhibitor therapy from basic research to FRDA clinical trials, the next few years will hopefully see the emergence of at least one effective epigenetic-based therapy for FRDA.

## Author contributions

All authors contributed to draft the manuscript and all authors read and approved the final manuscript.

### Conflict of interest statement

The authors declare that the research was conducted in the absence of any commercial or financial relationships that could be construed as a potential conflict of interest.
